# Women's Skin throughout Lifetime

**DOI:** 10.1155/2014/328981

**Published:** 2014-04-13

**Authors:** Gérald E. Piérard, Corinne Charlier, Philippe Delvenne, Philippe Humbert, Claudine Piérard-Franchimont

**Affiliations:** ^1^Laboratory of Skin Bioengineering and Imaging (LABIC), Department of Clinical Sciences, Liège University, 4000 Liège, Belgium; ^2^Department of Clinical Toxicology, University Hospital of Liège, 4000 Liège, Belgium; ^3^Department of Pathology, Unilab Lg, University Hospital of Liège, 4000 Liège, Belgium; ^4^Department of Dermatology, University Hospital Saint-Jacques, 25030 Besançon, France; ^5^Inserm U645 Research Unit IFR133, 25030 Besançon, France; ^6^Department of Dermatopathology, Unilab Lg, University Hospital of Liège, 4000 Liège, Belgium; ^7^Department of Dermatology, Regional Hospital of Huy, 4500 Huy, Belgium

The distinction between skin of men and women stems as much from biologic differences as from their different social roles and status in various societies. Currently, there is agreement among clinicians and researcher scientists to distinguish between different episodes in female life according to their hormonal status. They influence the skin physiology and the risk of diseases and possibly alter the quality of life. The present special issue contains ten papers focused on recent developments in the field of women's skin and mucosae. Two papers deal with physiological aspects of breasts prior to surgical intervention, as well as the skin response to seasonal environmental changes. One paper is focused on new developments in toxicological aspects related to selected hormone disruptors. Seven other papers focus on peculiar aspects of a series of skin disorders developed in women.

The paper entitled “*A methodological evaluation of volumetric measurement techniques including three-dimensional imaging in breast surgery,*” by H. Hoeffelin et al., describes objective and noninvasive methods for assessing breast volume. The 3D LifeViz system (Quantificare) was used in various settings (in situ on corpse dissection, on control prostheses, and in clinical conditions). Such a system was compared to other methods (CT scanning and Archimedes' principle) under the same conditions. The parameters of feasibility, safety, portability, minimal patient stress, and limitations (underestimation of the in situ volume, subjectivity of contouring, and patient selection) of the LifeViz 3D system indicate similar benefits compared to other measurement methods. The prospects of this method appear promising for a series of applications in clinical practice in order to limit the subjectivity of breast surgery.

The paper entitled “*The weather-beaten dorsal hand clinical rating, shadow casting optical profilometry, and skin capacitance mapping,*” by M. Delvenne et al., is an original work about a common condition linked to seasonal skin presentation associated with environmental changes. The withered skin surface changes were assessed during the four seasons. Among 47 menopausal women completing the study, 31 volunteers were on hormone replacement therapy (HRT) and 16 did not use HRT. Skin xerosis and scaliness were rated following clinical assessments. In addition, skin withering of the dorsal hands was assessed by computerized shadow casting optical profilometry. Skin capacitance mapping was also performed. Marked changes over the seasons were recorded corresponding to the combination of patchy heterogeneous stratum corneum hydration and heterogeneous skin surface roughness. These features likely resulted from variations in the environmental temperature and moisture. Daily stress due to alternate outdoor and indoor conditions was possibly involved in the skin condition. The severity of changes revealed by clinical inspection was not supported by similar directions of fluctuations in the instrumental assessments. Such contradiction was in fact due to different levels of scale observation. The clinical centimetric scale and the instrumental inframillimetric scale possibly provided distinct aspects of the biological impact.

The paper entitled “*Measurement of urinary biomarkers of parabens, phthalates, and benzophenone-3 in a Belgian population*,” by L. Dewalque et al., addresses the concerns about hormone disruptors. Phthalates, parabens, and benzophenone-3 (BP3) are commonly present in plasticizers, antimicrobial conservatives, and UV-filters, respectively. They exhibit endocrine disrupting properties yielding, for instance, to skin malignant melanoma. Humans are exposed to such chemicals through different sources like food, personal care preparations, and cosmetics. In this study, the exposure to five phthalates, four parabens, and BP3 was assessed by measuring urinary levels of their biomarkers in samples collected from 261 volunteers living in the Liège region of Belgium. The analyses were carried out by liquid chromatography tandem mass spectrometry (LC-MS/MS) with deuterated standards. The phthalate metabolites, BP3, and most of the parabens were detected in 82.8 to 100.0% of the samples. For most of these chemicals, the exposure patterns seemed to differ between children and adults. In addition, differences were perceived between males and females, especially with significantly higher concentrations of parabens in females. If a correlation was established between the incidence of some cutaneous pathologies, for example, malignant melanoma, and the presence of endocrine disrupting chemicals in biological samples, the present results are puzzling. Further studies on the subject are necessary to clarify this relationship.

In the paper entitled “*Pruritus in female patients*,” JMRG Lambert reviews the global understanding about pruritus affecting some women. Pruritus is a frequent symptom in a number of skin diseases. This review was focused on specific itch problems specific to women, namely, pruritic vulvar dermatoses, and specific pruritic dermatoses of pregnancy. The characteristics of the vulva and the hormonal changes during the different age periods make these dermatoses very particular. It seems that vulvar diseases remain underdiagnosed and undertreated. Pruritic vulvar diseases have a severe impact on quality of life. The most common pruritic diseases concern atopic and contact dermatitis, psoriasis, lichen sclerosus, lichen planus, and infectious vulvovaginitis. The diagnostic issues of these diseases are discussed and the general principles of therapy are covered.

In the paper entitled “*The female pattern hair loss in 2013: Review of etiopathogenesis and diagnosis,*” A. Vujovic and V. Del Marmol review concepts regarding female pattern hair loss (FPHL), representing the most common hair loss disorder in women. Initial signs possibly develop in teenagers leading to a progressive hair loss with a characteristic distribution pattern. The condition is characterized by progressive replacement of terminal hair follicles over the frontal and vertex regions by miniaturized follicles. This evolution leads progressively to a perceptible reduction in hair density. Women diagnosed with FPHL may undergo significant impairment of quality of life. FPHL diagnosis is mostly clinical. Depending on patient history and clinical evaluation, further diagnostic testing is occasionally useful.

In the paper entitled “*Female gender and acne disease are jointly and independently associated with the risk of major depression and suicide: a national population-based study,*” Y. C. Yang et al. address the association of acne, poor self-esteem, and social phobia in Taiwanese people. Previous studies based on questionnaires from several thousand adolescents had showed that acne was particularly associated with major depression and suicide. Using a 2006 database from the National Health Insurance in Taiwan, patients with acne, major depression, and suicide were considered. A total of 51689 patients with acne were identified (17974 males and 33715 females) from 1 million subjects. The youths (7–12 years) had the highest prevalence of acne (15.87%). Major depression was more common in patients with acne (0.76%) than in controls (0.56%, *P* < 0.0001) regardless of gender. Multiple logistic regressions showed an increased risk to major depression in women without acne. The risk was further increased in women with acne. Similar increased risk of suicide was noticed in acneic women. In conclusion, acne and gender, independently and jointly, are associated with major depression and suicide. Special medical support should be warranted in females with acne for the risk of major depression and suicide.

The paper entitled “*Psoriasis: female skin changes in various hormonal stages throughout life*—*puberty, pregnancy, and menopause,*” by R. Ceovic et al., discusses the fate of psoriasis in women. Stress greatly affects both the hormone and immune systems. The severity of psoriasis often fluctuates or is influenced with each hormonal phase in women and this relationship affects some disease frequencies peaking at puberty, during postpartum, and menopause, while symptoms improve during pregnancy.

The paper entitled “*Vulvar skin disorders throughout lifetime: main dermatoses,*” by J. Doyen et al., addresses four representative vulvar dermatoses. Lichen simplex chronicus is a pathological condition related to friction. Such detrimental effects in the presence of other dermatoses have to be considered when therapeutic responses are unsatisfactory. Lichen sclerosus is a common vulvar dermatosis particularly in the elderly. Lichen planus is a distinct entity. Paget's disease, although a rare condition, represents a clinical and therapeutic challenge.

The paper entitled “*A clinical and pathological overview of vulvar condyloma acuminatum, intraepithelial lesions, and squamous cell carcinoma,*” by B. Léonard et al., is focused on some vulvar epithelial neoplasms. Condyloma acuminatum, intraepithelial neoplasia, and squamous cell carcinoma are three common vulvar lesions. Condyloma acuminatum is induced by low risk genotypes of human papillomavirus (HPV). Vulvar intraepithelial neoplasia (VIN) and squamous cell carcinoma have different etiopathogenic pathways and are related or not with high risk HPV types. This article reviews the main pathological and clinical features of these lesions. A special attention is paid to epidemiology, pathological classification, and clinical implications of these diseases.

The paper entitled “*Streamlining cutaneous melanomas in young women of the Belgian Mosan region*,” by T. Hermanns-Lê and S. Piérard, is a review article focused on sporadic cutaneous melanomas (SCM) developing in women during their childbearing age. This neoplasm has shown an increased incidence over the past few decades. The vast majority of these SCM were of the superficial type without any obvious relationship with a large number of melanocytic nevi. Signs of frequent and intense sunlight exposure were not disclosed by the extent in the mosaic subclinical melanoderma. A series of investigations point to a possible relationship linking the development of some of these SCM with the women hormonal status including the effect of hormonal disruptors. These aspects remain, however, unsettled and controversial. It is possible to differentiate and clearly quantify the SCM shape, size, scalloped border, and variegated pigmentation using computerized morphometry as well as fractal and multifractal methods.

We hope this special issue will not only help to understand and diagnose some skin disorders typically found in women, but also reinforce the development of better management modalities for these women skin disorders.

*Gérald E. Piérard* *Corinne Charlier* *Philippe Delvenne* *Philippe Humbert* *Claudine Piérard-Franchimont*

